# Interactions and ultrafast dynamics of exciton complexes in a monolayer semiconductor with electron gas

**DOI:** 10.1515/nanoph-2023-0913

**Published:** 2024-02-05

**Authors:** Aleksander Rodek, Kacper Oreszczuk, Tomasz Kazimierczuk, James Howarth, Takashi Taniguchi, Kenji Watanabe, Marek Potemski, Piotr Kossacki

**Affiliations:** Faculty of Physics, University of Warsaw, ul. Pasteura 5, 02-093 Warszawa, Poland; National Graphene Institute, University of Manchester, M13 9PL, Manchester, UK; International Center for Materials Nanoarchitectonics, National Institute for Materials Science, 1-1 Namiki, Tsukuba 305-0044, Japan; Research Center for Functional Materials, National Institute for Materials Science, 1-1 Namiki, Tsukuba 305-0044, Japan; Laboratoire National des Champs Magnétiques Intenses, CNRS-UGA-UPS-INSA-EMFL, 25 Av. des Martyrs, 38042 Grenoble, France; CENTERA Labs, Institute of High Pressure Physics, PAS, 01-142 Warszawa, Poland

**Keywords:** nonlinear spectroscopy, transition metal dichalcogenide monolayer, Fermi sea, exciton, trion, ultrafast dynamics

## Abstract

We present femtosecond pump-probe measurements of neutral and charged exciton optical response in monolayer MoSe_2_ to resonant photoexcitation of a given exciton state in the presence of 2D electron gas. We show that creation of charged exciton (X^−^) population in a given K^+^, K^−^ valley requires the capture of available free carriers in the opposite valley and reduces the interaction of neutral exciton (X) with the electron Fermi sea. We also observe spectral broadening of the X transition line with the increasing X^−^ population caused by efficient scattering and excitation induced dephasing. From the valley-resolved analysis of the observed effects we are able to extract the spin-valley relaxation times of free carriers as a function of carrier density. Moreover, we analyze the oscillator strength and energy shift of X in the regime of interaction with electron Fermi sea under resonant excitation. From this we can observe the process of X decay by radiative recombination paired with trion formation. We demonstrate an increase of neutral exciton relaxation rate with the introduction of Fermi sea of electrons. We ascribe the observed effect to the increased efficiency of the trion formation, as well as the radiative decay caused by the screening of disorder by the free carriers.

## 1 Introduction

Single layers of semiconducting transition metal dichalcogenides (sTMDs) display a plethora of intriguing optical, electronic and valley properties which have driven large interest over the previous decade [1], [2], [3], [4], [5]. MoSe_2_ is unique among sTMDs as the lowest available transition is also spin allowed [3], [6], [7]. The neutral exciton (X) resonance in this material is accompanied by a ∼30 meV split trion or charged exciton (X^−^) state with their relative amplitudes depending on the carrier density, which may be influenced by a number of factors like the density of charged defects, hBN encapsulation or the properties of the optical excitation [8], [9], [10]. Large oscillator strengths (osc. str.) of these transitions also facilitate studies of the dynamics of nonlinear effects; however, due to the short exciton lifetimes of a few ps, these attempts require ultrafast temporal resolutions [11], [12], [13], [14], [15], [16], [17]. Optically generated nonlinearities of neutral exciton transition, like bleaching, energy shifts and excitation induced dephasing (EID) were observed, at least for the case of naturally doped samples [17], [18], [19], [20], [21], [22]. Recently, many studies have turned towards probing the exciton-carrier interactions. By means of electron beam litography one can manufacture electrodes in the vicinity of TMD flakes allowing for controlled tuning of the carrier density by the application of an external voltage [23], [24], [25]. This approach has led to milestone results in the field of strongly-correlated electron systems: like Wigner crystallization [26], optical sensing of the quantum Hall effect in graphene [27] and tunable quantum confinement of neutral excitons [28]. Such carrier injection also directly influences the observable exciton transitions. Following the nomenclature coined in atomic physics, the charged and neutral exciton states are often described as attractive and repulsive polarons [29]. The experimental observations, as well as theoretical modeling of the interaction of excitons in TMDs with free carriers point to pronounced changes of exciton energy, linewidth and oscillator strength [23], [24], [30], [31], [32], [33], [34] similarly to what has been observed for the traditional 2D systems of II–VI [35], [36], [37], [38], [39] and III–V [40], [41], [42] quantum wells. Theoretical works also point to a significant influence of carriers on the specific relaxation dynamics of excitons [43]. In particular, recent experimental studies on MoTe_2_ [44] show a significant prolongation of the valley polarization for larger densities of free carriers. This is also quite relevant in the context of the efficient exciton-trion formation channel discovered in the naturally doped samples [9], which may be partly responsible for the observable increase of the exciton’s homogeneous linewidth with increasing carrier density [45], [46]. Here, we consider these issues by focusing on the exciton absorption, while capitalizing on the high temporal resolution of ultrafast pump-probe measurements on a charge-tunable monolayer (ML) MoSe_2_ sample. Incidence angle separation between the laser beams, which facilitates a high degree of filtering of the pump signal, allows us to perform measurements of the response of X and X^−^ transitions after selective excitation of a given state. Measurements performed for different configurations of circular polarizations probe the intra-/inter-valley exciton dynamics and the influence of both valley occupations on the excitonic absorption. In particular we show that the effects observed under selective excitation of the K-valley charged exciton can be explained by the capture of K′-valley electron leading to an effective change of the free carrier density in one of the valleys and subsequent screening of exciton interactions with the 2D carrier gas. Here we are also able to pinpoint the effect of the neutral exciton dephasing induced by the population of photocreated charged excitons further elucidating the importance of coupling between these states. Time-resolved measurements in the circular polarization basis allow us to probe the exciton valley and population dynamics in the first few ps and investigate their dependence on the increasing density of electron gas.

## 2 Methods and sample characterization

**Figure 1:**
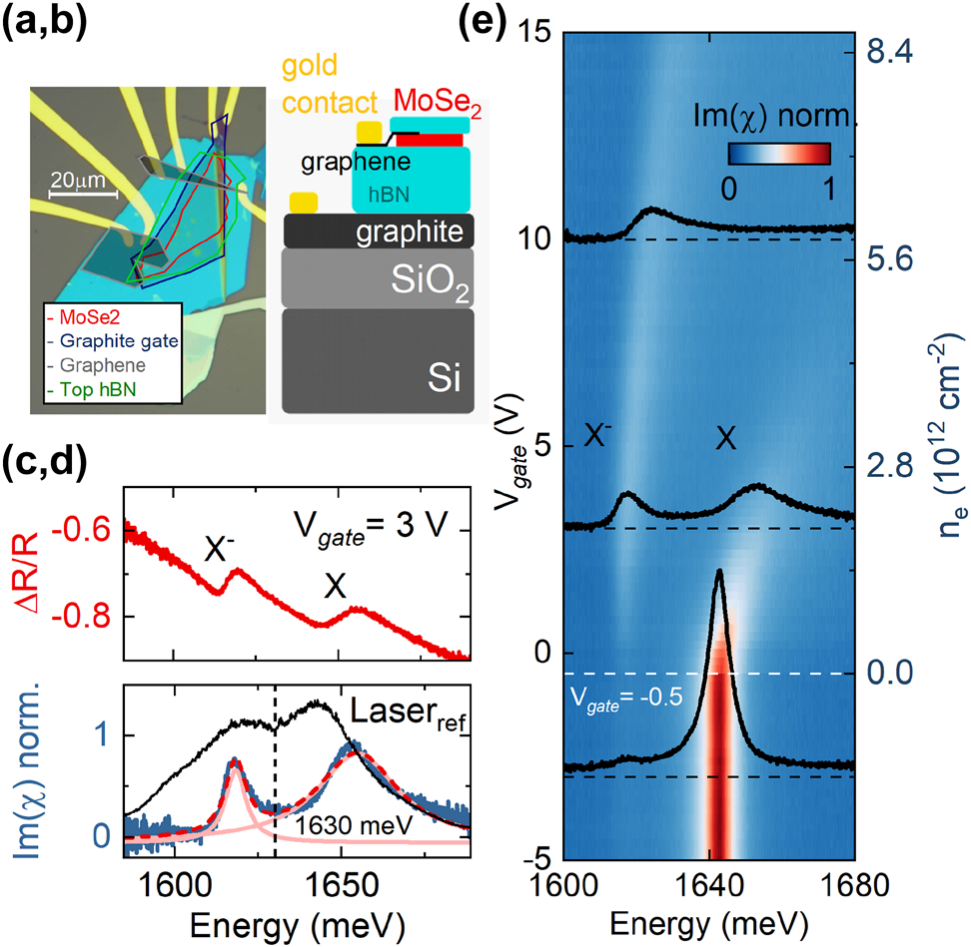
Sample characterization sample optical image (a) and heterostructure schematic (b). (c) Differential reflectance of the sample for *V*_*gate*_ = 3 V with visible charged and neutral exciton resonances. (d) Normalized imaginary susceptibility obtained from Kramers–Kronig transformation of the spectra in (c) with the reference fs laser spectrum. Dashed line indicates the cut-off energy for short-/long-pass filters used for resonant neutral/charged exciton excitation. (e) Electric scan of the X and X^−^ signal for *V*_*gate*_=(−5:15)V Dashed line indicate the charge neutrality point of the sample.

In Figure 1(a–b) we present an optical image of the sample as well as the schematic of the heterostructure. It consists of a single layer MoSe_2_ that was encapsulated between hBN flakes. Their thicknesses deduced from atomic force microscope measurements are hBN_bottom_ = 34.5 nm and hBN_top_ ≈ 5 nm. In our case a graphite flake located below the thick hBN spacer acts as a bottom gate, while the top contacts are made out of few-layer graphene flakes deposited directly on the monolayer. The graphene/graphite flakes are connected to golden contacts prepared by e-beam lithography. We optically characterize the device by measuring reflectance of a broad fs laser (spectral full-width at half maximum FWHM ≈ 40 meV) tuned between the neutral and charged exciton transitions (*E* ≈ 1642 meV, Figure 1(d)) in low temperature (*T* = 5 K). In the Figure 1(c) we show a differential reflectance spectrum of the sample ((*R*_*sample*_–*R*_*ref*_)/*R*_*ref*_) with the applied gate voltage of *V*_*gate*_ = 3 V in order to present both exciton resonances. By applying the Kramers–Kronig transformation [20], [47], we obtain the imaginary part of sample’s susceptibility and disregard the interference patterns leading to the complex lorentzian shape of the exciton lines (Figure 1(c–d)). To characterize our sample as a function of free carrier density we tune the *V*_*gate*_ between −5 V and 15 V and present the obtained spectra in Figure 1(e). The density of the free electrons induced by changing the gate bias was calculated from a simple planar capacitor model [23], [48] and equaled *n*_*e*_ = 5.6∗10^11^ cm^−2^ per 1 V. By changing the gate voltage we observe the expected response of the neutral exciton peak to the increasing carrier density: (i) Transfer of the osc. str. from X to X^−^, (ii) Increase of the X energy, (iii) Broadening of the X peak.

**Figure 2:**
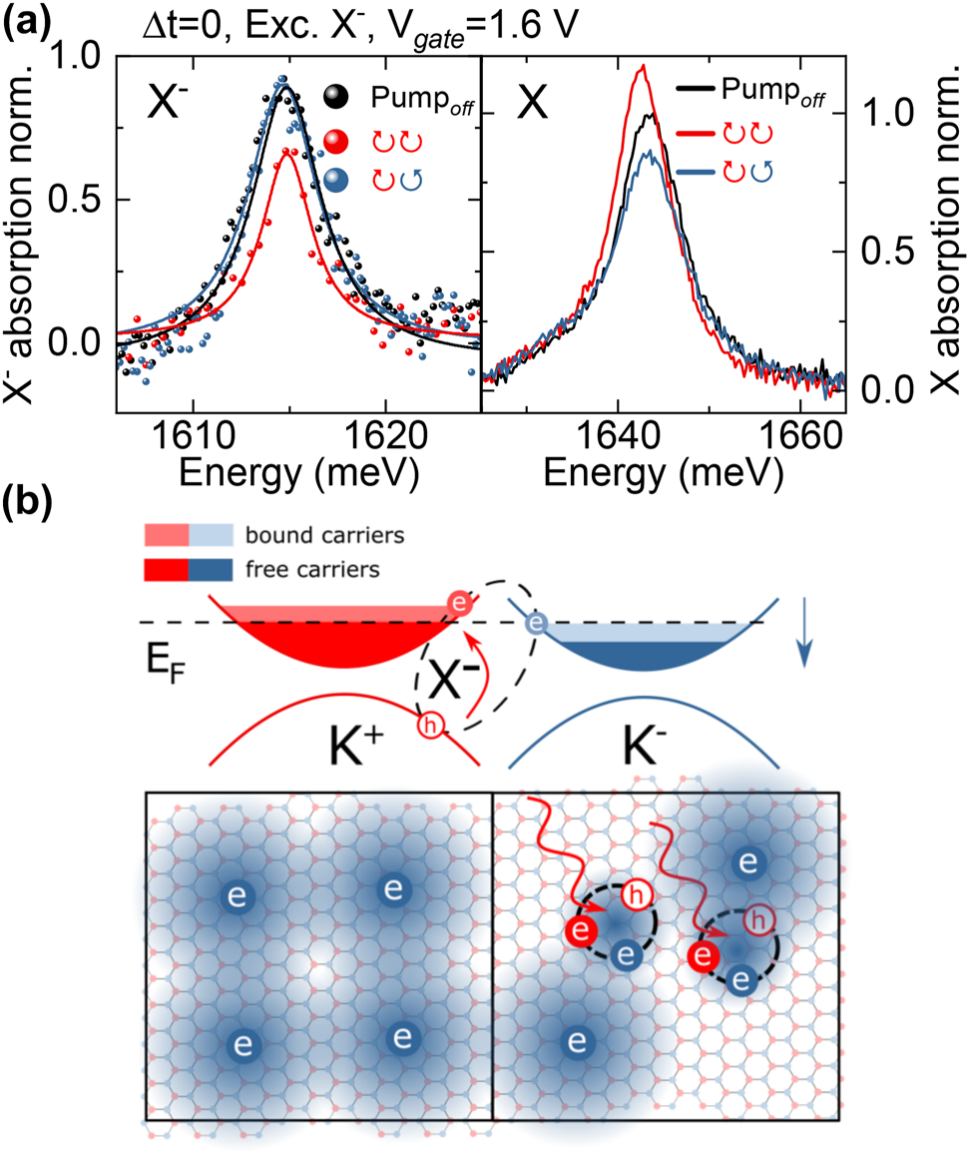
Polarization-resolved spectra of X and X^−^ under X^−^-resonant excitation. (a) Neutral and charged exciton spectra with/without X^−^ excitation in the coincidence (Δ*t* = 0) in a given circular polarization configuration. *V*_*gate*_ = 1.6 V, *n*_*e*_ = 11.8∗10^11^ cm^−2^, photocreated density of $n_{X^{-}}\approx 2*10^{11}$ cm^−2^. (b) Drawing of the influence of X^−^ creation in a K^+^ valley on the population of free carriers in K^−^ valley. In the Fermi sea regime of carrier density the photocreated population of X^−^ binds with the available free carriers effectively lowering electron density in the opposite valley.

The pump-probe measurements presented in this study were performed in a back-reflection geometry [20]. The beams were focused onto the sample surface through a short focal-length lens (*f* = 4 mm) with the spot diameter of the probing beam of *d* = 3.8 μm. This is particularly important for the case of measurements of TMD heterostructures manufactured through exfoliation-based methods as they near-universally lead to significant spatial disorder. This affects the exciton resonances, leading to large inhomogeneous broadening. Microspectroscopy allows for the reduction of such influence by probing a smaller sample area. The optical paths of the pump and probe laser beams were separated by a small angle permitting us to distinguish between the beams in the detection path. Both of the laser beams had the same spectral shape centered between the exciton resonance. In the following experiments we used long- and short-pass interference filters to select the pumping energies to either above or below the energy of 1630 meV, which allowed for the resonant excitation of either neutral or charged exciton states. The temporal resolution of our setup was limited by the time duration of the pulses *σ*_*t*_ ≈ 80 fs at the sample surface.

## 3 Selective excitation of X^−^

We start our analysis with the simplest case of selective excitation of X^−^. The charged exciton is the energetically-lowest state therefore the relaxation considered here is less involved.

Firstly, we discuss the results obtained at pulse overlap Δ*t* = 0. We set the gate voltage to 1.6 V which corresponds to a relatively low-doping regime with *n*_*e*_ = 11.8∗10^11^ cm^−2^, however with already visible effects of carrier gas impact: oscillator strength transfer (significant X^−^ absorption) and energy shifts (neutral exciton blueshift of a few meV). In Figure 2(a) we present the optical response of neutral and charged excitons for different polarization configurations (co-/cross-circular) with the pumping beam resonant with the charged exciton. The pump pulse induces pronounced reduction of the charged exciton oscillator strength in the co-polarized case with no visible change of this state in the opposite valley. This can be understood when considering the charged exciton as a bound three particle complex of two electrons from opposite K^±^ valleys and a hole. As we create a population of K^+^ charged excitons, the free carriers from the K^−^ valley become bound. This in turn reduces the available density of free electrons for the creation of a K^+^ charged exciton. This process is illustrated in Figure 2(b). Conversely the population of free electrons in K^+^ valley does not change and as such the K^−^ charged exciton remains mostly unaffected.

We now consider the response of the neutral exciton when pumping charged exciton. In the case where we excite X^−^ in the same K^+^ valley the X resonance exhibits a redshift of ∼1 meV, an increase of its oscillator strength and a linewidth narrowing. Again, an explanation of these effects can be provided by considering the density of free carriers in the opposite K^−^ valley. As the K^+^ charged and neutral excitons share a common ground state, upon the illumination of the sample a photon of a *σ* ^+^ polarization can be converted to either of these states with the probability ratio depending on the availability of free carriers in the opposite K^−^ valley. This results in the oscillator strength stealing from the neutral to charged exciton in K^+^ valley if the number of K^−^ free carriers increases.

Reduction of K^−^ free carrier density through the photocreation of K^+^ charged excitons quenches the Fermi sea-induced effects upon the neutral exciton state and results in the increased oscillator strength, as well as an effective redshift and decreased linewidth. Interestingly, similar phenomena have also been reported for standard QWs [49], [50].

The neutral exciton in the opposite valley shows no energy change. This is understandable as the free carrier density in the K^+^ valley is not influenced by the creation of a K^+^ charged exciton. The only observable effect is its linewidth broadening, which cannot be explained by considering solely the free electron population. Consequently any changes in the K^−^ neutral exciton signal must be related to the population of photocreated bound carriers forming the charged excitons. Therefore we point out that the resulting linewidth broadening can be considered as an excitation induced dephasing (EID) of neutral exciton by the population of charged excitons. This effect has also been previously reported in works that revealed coherent coupling between these states [51], [52], however, it has not been investigated in greater detail.

In order to provide a more quantitative description of this effect we performed power dependent measurements of neutral exciton absorption with resonant charged exciton excitation.

**Figure 3:**
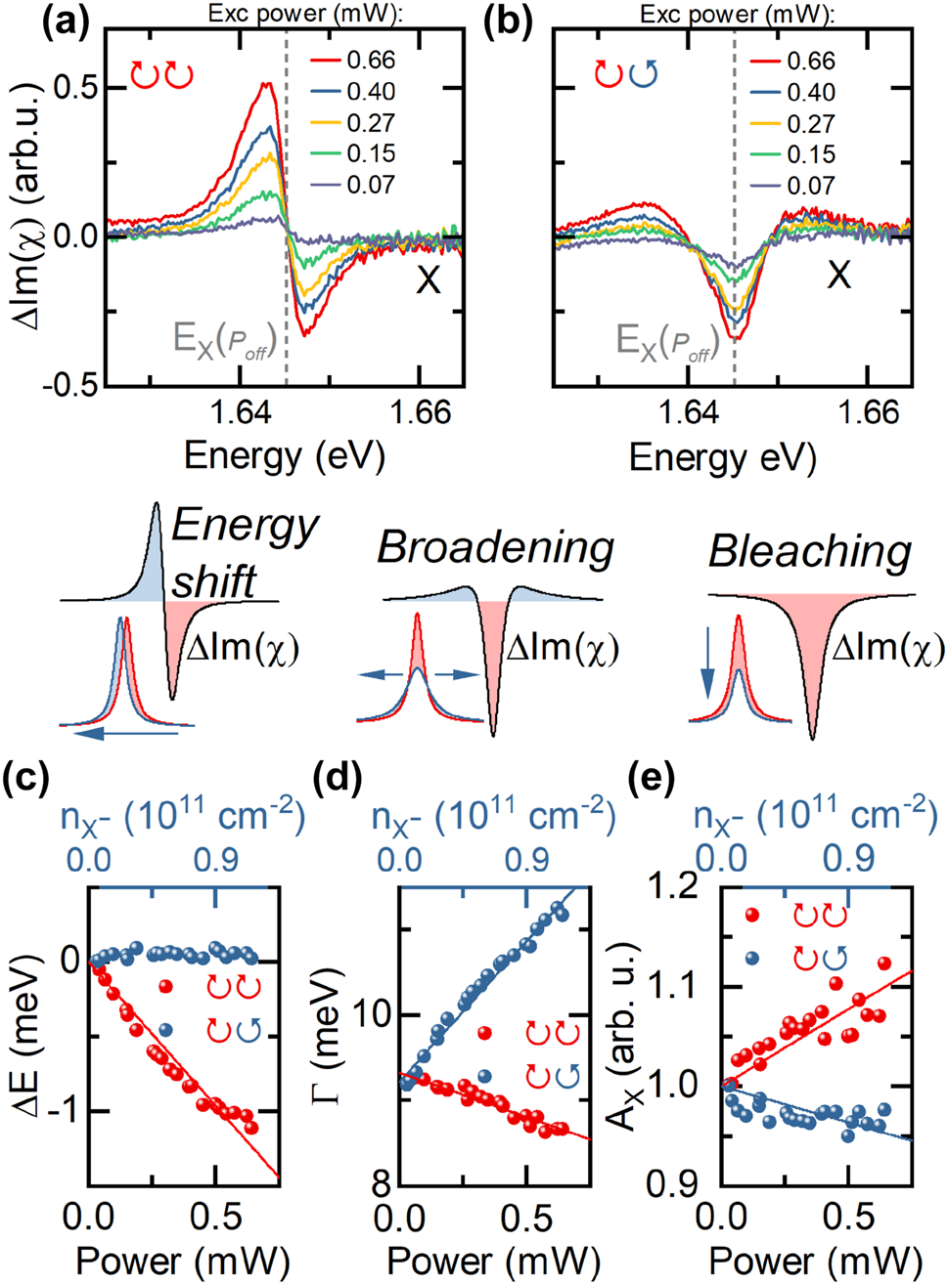
Power dependence of neutral X absorption while under resonant X^−^ excitation (Δ*t* = 0), *V*_*gate*_ = 1 V, *n*_*e*_ = 8.4∗10^11^ cm^−2^ X differential absorption in co-(a) and cross-(b) polarized configuration under resonant X^−^ excitation as a function of pump power. (c–e) X energy shift, linewidth and oscillator strength in the co- and cross-polarized detection under resonant X^−^ excitation as a function of excitation power. Solid lines indicate linear fits to the data.

In Figure 3(a, b) we present the difference in the neutral exciton signal induced by the charged exciton illumination versus the power of the pump laser beam. In the co-circular excitation case we observe the dominating effect of energy shift as well as the additional linewidth narrowing and osc. str. increase. For the case of the cross-polarized excitation we see the pronounced broadening of exciton linewidth without any changes in its energy.

Quantitative determination of the behavior of the resonance parameters was performed by fitting a Lorentzian function to the X absorption signal. We show the fitted values in Figure 3(c–e). In all related cases we find a linear dependence of the extracted parameters on the excitation power and thus the density of photocreated charged excitons. This confirms that we operate far from the saturation regime.

For the energy of the neutral exciton the only change is observed in the co-circular excitation scheme. Its dependence on the free carrier density can be quantified through an effective parameter *η*:

(1) $$\Delta E_{X}^{\pm }=\eta *n_{e}^{\mp }$$

Since the photocreation of a charged exciton requires a capture of a free carrier the remaining free carrier density is given by $n_{e}^{\pm }=n_{\mathrm{gate}}^{\pm }-n_{X^{-}}^{\mp }$ , where *n*_*gate*_ is the initial density of carriers induced by the gate bias. From the Figure 3(c) we extract *η* = (0.8 ± 0.3)∗10^−11^ meV cm^2^. The value of *η* can be also independently derived from the exciton blueshift in the gate-dependent reflection measurement where $n_{X^{-}}$ = 0. In this case we obtain *η* = (1.3 ± 0.1)∗10^−11^ meV cm^2^. While the similarity of these values gives further evidence to the proposed interpretation of the observed phenomena, where optical control of carrier density can be achieved by resonant photocreation of charged excitons, small discrepancies are expected. This is because in contrast to the situation where the gas density is controlled only by the gate, reducing electron density by binding it in charged excitons still leaves such bound electrons in the band. Furthermore, the extracted trends persist for different gate biases (see Supplementary Material) indicating a constant magnitude of the many-body interaction strength in the investigated doping range.

Similarly, in the Figure 3(d) we present the values of neutral exciton linewidth Γ and its dependence on the photocreated density of X^−^. For the co-polarized case, the linewidth narrowing scales as ΔΓ_*X*_ = (−0.4 ± 0.1)∗10^−11^ meV ${cm}^{2}*n_{X^{-}}$ . For the exciton in the opposite valley, the linewidth increases as ΔΓ_*X*_ = (1.4 ± 0.3)∗10^−11^ meV ${cm}^{2}*n_{X^{-}}$ , reflecting the EID by the population of charged excitons in the opposite valley.

Accordingly to the proposed interpretation these effects of linewidth narrowing/broadening in the co/cross-polarized excitation scheme also persist for the entire range of investigated bias values (see Supplementary Material). Here, a more in-depth analysis is, however, obstructed by the presence of inhomogeneous addition to the exciton linewidth. Previous four-wave-mixing studies [45], [46], which are able to independently extract the homogeneous and inhomogeneous linewidth, show, that increasing density of free carriers leads to increasing homogenenous broadening, related to the shortening of exciton coherence time, as well as the screening of disorder and decreasing inhomogeneous broadening. In our case this leads to an effective broadening of exciton resonance as we increase the gate bias ΔΓ_*X*_ = (0.8 ± 0.1)∗10^−11^ meV cm^2^∗*n*_*e*_. Similar magnitudes of exciton linewidth changes induced by the gate injection of free electrons and by their capture through charged excitons creation again point to the same origin of these effects, as was the case for the considerations of exciton energy shifts.

As previously mentioned, the neutral exciton total absorption also changes, particularly for the case of co-polarized excitation as it displays a pronounced increase. Its dependence on the density of photocreated X^−^ is presented in the Figure 3(e). This behavior can be quantitatively described as:

(2) $$\Delta A_{X}^{\pm }=-A_{X}\left(0\right)\left[\alpha *n_{e}^{\mp }+\beta \left(n_{X^{-}}^{+}+n_{X^{-}}^{-}\right)\right]$$

where *A*_*X*_(0) – neutral exciton osc. str. in the neutrality regime used here as a normalization parameter, *α*, *β* – effective parameters quantifying the change in the X absorption due to the population of free carriers and photocreated charged excitons. From linear fitting shown in the Figure 3(e) we determine *α* = (6.3 ± 1.4)∗10^−13^ cm^2^ and *β* = (2.8 ± 0.6)∗10^−13^ cm^2^

Again the gate-dependent measurement give a consistent value of *α* = (5 ± 0.2)∗10^−13^ cm^2^. In the Supplementary Figure S1 we also present the behavior of charged- and neutral exciton total absorption, illustrating the effect of oscillator strength transfer.

### 3.1 Time-resolved pump-probe with selective excitation of X^−^

**Figure 4:**
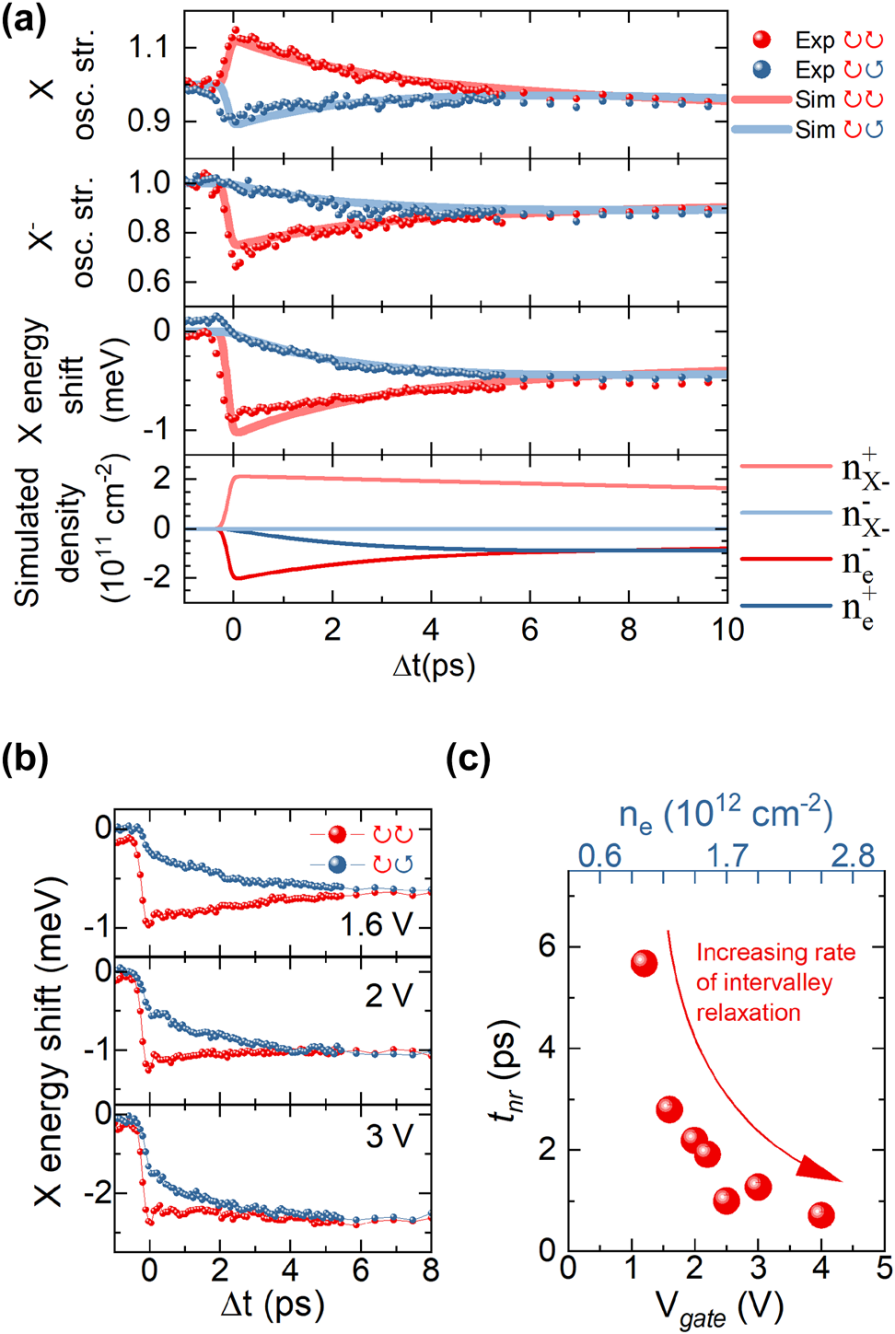
X, X^−^ dynamics under X^−^-resonant excitation (a) valley-resolved X, X^−^ amplitude and X energy shift dynamics under X^−^-resonant excitation in the first few ps. Solid lines denote values obtained from the simulations. Simulated population of photocreated charged excitons and related change in the free carrier density. V_*gate*_ = 1.6 V (b) valley-resolved dependence of the X redshift time evolution for different gate voltages. (c) Extracted spin-valley relaxation times of free carriers as a function of gate voltage.

In order to investigate the ultrafast dynamics we have performed the pump-probe measurements with a variable delay Δ*t* between the laser pulses. In the Figure 4(a) we present the time evolution of neutral and charged exciton oscillator strengths and neutral exciton energy in the pump-probe measurement.

For Δ*t* = 0 we observe the previously mentioned changes in the integrated osc. str. of the resonances, as well as the neutral exciton energy redshift. The most striking effect occurs in the first few ps where we see a rapidly decreasing difference in the polarization dependence of the observed effects with a single characteristic time of *t*_*nr*_ = (2.7 ± 0.2) ps.

In order to gain further understanding of the measured dynamics we develop a rate equations approach that considers a change in population of free carriers $n_{e}^{\pm }$ by photocreation of charged excitons $n_{X^{-}}^{\mp }$ in the opposite K-valley. The dependence of neutral exciton energy and osc. str. on the simulated carrier populations is described by the equations (1) and (2). Additionally, we introduce another parameter *γ* governing the change of the charged exciton oscillator strength:

(3) $$\Delta A_{X^{-}}^{\pm }=A_{X}\left(0\right)\left[\gamma *n_{e}^{\mp }\right]$$

In our model we include two decay mechanisms important for the dynamics of X^−^:

– the charged exciton decay with characteristic time $t_{X^{-}}$ = 40 ps, which was independently extracted from the time-resolved photoluminescence measurement on a streak camera (see Supplementary Material)
– the intervalley scattering of free carriers *t*_*nr*_.

The following set of rate equations was used for producing the simulations of pump-probe experiments presented in the Figure 4(a).

(4) $$\frac{dn_{X^{-}}^{\pm }}{dt}=\Theta_{\mathrm{Laser}}^{\pm }\left(t\right)-\frac{n_{X^{-}}^{\pm }}{t_{X^{-}}}$$

(5) $$\frac{dn_{e}^{\pm }}{dt}=-\Theta_{\mathrm{Laser}}^{\mp }\left(t\right)-\frac{n_{e}^{+}-n_{e}^{-}}{t_{nr}/2}+\frac{n_{X^{-}}^{\mp }}{t_{X^{-}}}$$

where $\Theta_{\mathrm{Laser}}^{\mp }$ – excitation laser intensity given by a Gaussian pulse, $n_{X^{-}}^{\pm }$ – population density of charged excitons, $n_{e}^{\pm }$ – change of the free carrier population with respect to the net density at the particular gate bias (at V_*gate*_ = 1.6 V $n_{e}^{0}=11.8*10^{11}$ cm^−2^), $t_{X^{-}},t_{nr}$ – relaxation time of charged exciton and intervalley scattering time of free carriers. Equation (1) governs the evolution of charged excitons after their resonant creation by the pump pulse. Equation (2) describes the changes in the free carrier density induced by the creation of charged excitons. The simulated populations are presented in Figure 4(a).

The outcome of the simulation is plotted as solid lines in the Figure 4(a) illustrating an excellent agreement with the data and confirming a dominant role of free carriers in the exciton dynamics after resonant creation of charged excitons.

In particular, within the proposed model the differences in the co-/cross-polarized signals directly reflect the change in the valley populations of unbound carriers. As the trion consists of two electrons from both K-valleys and one hole, its intervalley scattering does not affect the electron populations and in principle could be taking place at a different timescales.

We also investigate the intervalley scattering process as a function of the gate voltage. In the Figure 4(b) we plot the neutral exciton energy shifts in co-/cross-polarization configurations for selected gate voltages. It shows a pronounced shortening of the scattering time for higher electron densities, which can be easily extracted by fitting the decay of the difference in exciton energy redshifts with an exponential function. The obtained decay times are shown in the Figure 4(c). We observe an order of magnitude reduction of this scattering time starting from around 6 ps for low electron density of 10^12^ cm^−2^ to 0.7 ps measured at 2.5∗ 10^12^ cm^−2^ carrier density. This is particularly interesting in the context of previous studies on this process for various TMD systems [53], [54], [55], [56], [57], [58], [59]. While most of these works focused on other TMD materials, which differ from MoSe_2_ with their particular band configuration and the optical activity of the ground exciton state, the ones that explored the dependence of the spin-flip process on electrostatic doping consistently show that its characteristic timescale decreases with the increasing free carrier density. Only difference are the reported intervalley scattering times of resident carriers, which were in the order of hundreds of ns. Furthermore, a recent report, which directly probed the carrier relaxation dynamics in MoSe_2_ occupying the Landau quantized states in high magnetic field, also shows a similarly slow process [60]. Interestingly, this work provides strong evidence for the increased efficiency of the resident electron spin-flip process induced by the presence of neutral excitons. While this could partially explain such rapid scattering rates obtained in our study (if we assume that the pumping beam overlaps some part of the low-energy tail of neutral exciton absorption) it also implies that the measured values would correspond to neutral exciton intervalley scattering times. However such an assumption directly contradicts previous works that showed instead timescales of hundreds of fs [20], [61]. Alternatively, we may consider that a similar process of valley-depolarization of carriers can be mediated by the charged exciton complexes. In this scenario, the observed timescales would be related to the intervalley scattering of bound carriers i.e. X^−^. This interpretation is further fortified by the fact that previous works reported similarly short ps valley depolarization times of trions in the naturally doped samples [61].

## 4 Selective excitation of X

**Figure 5:**
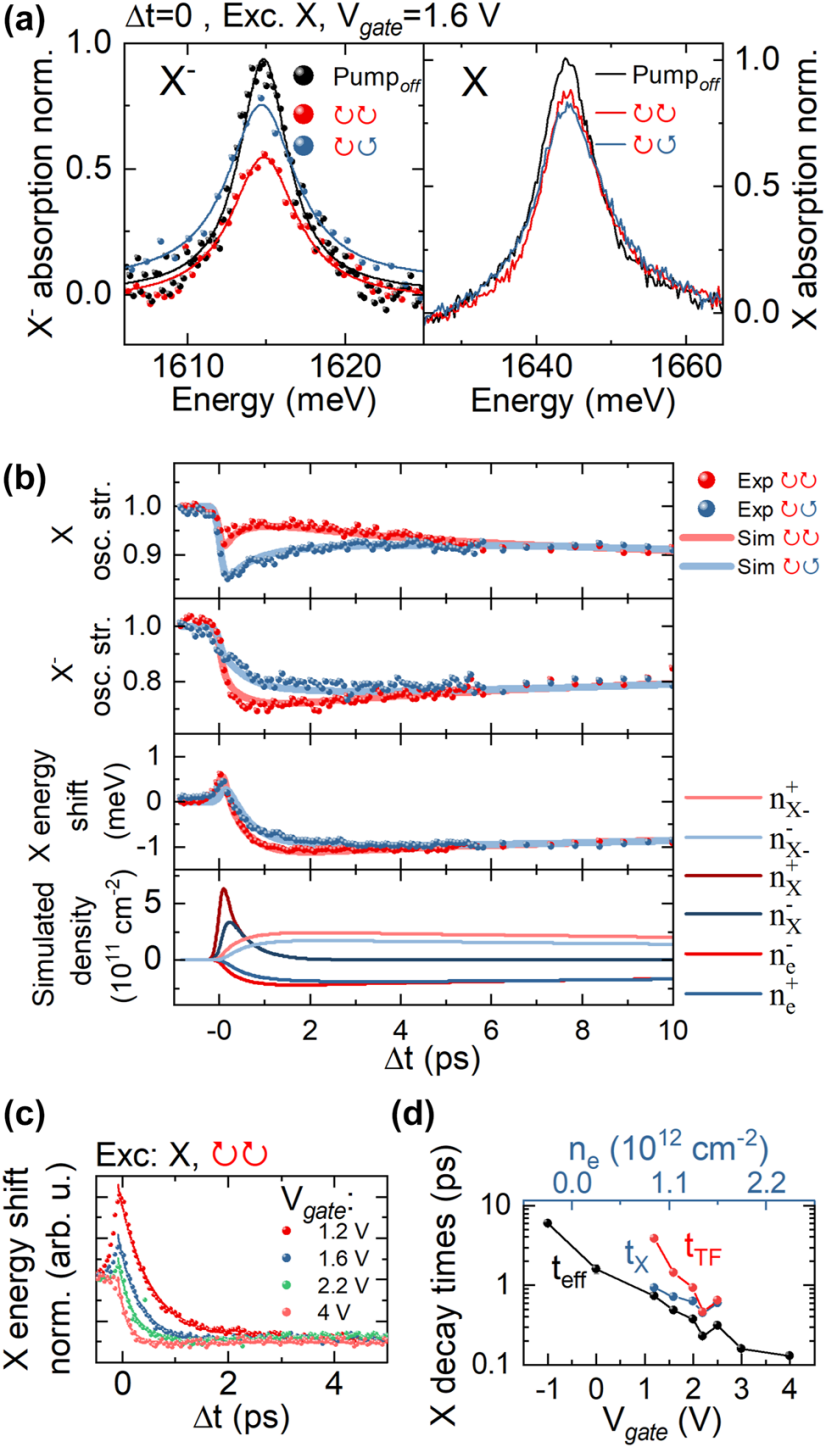
X, X^−^ dynamics under X-resonant excitation(a) neutral and charged exciton spectra with/without X excitation in the coincidence (Δt = 0) depending on the circular polarization configuration. V_*gate*_ = 1.6 V (b)Valley-resolved X, X^−^ amplitude and X energy shift dynamics under X-resonant excitation in the first few ps. Solid lines denote values obtained from the simulations. Simulated population of photocreated excitons and related change in the free carrier density. V_*gate*_ = 1.6 V (c) normalized X redshift dynamics under X-resonant excitation for different gate voltages with fitted exponential decay. (d) Extracted relaxation times of neutral X as a function of gate voltage.

In this section we present the investigation of exciton dynamics after resonant driving of the neutral exciton. In Figure 5(a) we show the change in excitons spectra at zero delay for *V*_*gate*_ = 1.6 V. The neutral exciton shows a slight blueshift, as well as the apparent bleaching of its total amplitude. These effects are similar, although present in a smaller scale, to what is observed in the non-doped regime without free carriers and is resultant of the exciton-exciton interactions (Figure S2, [20]). For the charged excitons we see an apparent decrease in its signal for both K-valleys. This can be again attributed to the influence of neutral exciton population by means of the excitation induced dephasing on the charged exciton. It is also a demonstration of the previously reported efficient coupling between these quantum states [45], [51], [52], [61], [62].

In principle, the cross-circularly polarized configuration of the pump-probe experiment may facilitate the observation of biexciton states, even for the case in which they are not visible in direct reflection measurements. While some works [62] indicate the presence of the biexciton transition line in MoSe_2_ monolayers with the binding energy of ∼20 meV, we do not observe any presence of such additional resonances in our case, even while changing the gate bias. We therefore omit the biexciton states in further considerations.

In Figure 5(b) we show the time evolution of exciton oscillator strengths and the neutral exciton energy in the pump-probe measurement. In the co-polarized configuration we observe that after the initial decrease of the neutral exciton oscillator strength it exhibits a slight increase in the first ps. This is accompanied by the transition of its energy from the initial blue-shift to a red-shift (Δ*E* ≈ 1 meV), and a large decrease of the charged exciton oscillator strength. Such dynamics is a fingerprint of the charged exciton formation process, where the neutral exciton captures an electron from the opposite valley thus lowering the free carrier density, while affecting the absorption spectra in a similar way as for the already discussed case of direct photocreation of charged excitons. The non-equilibrium population of free electrons relaxes then to the opposite valley with the same timescale of *t*_*nr*_ = (2.7 ± 0.2) ps as in the Figure 4(a), which results in the diminishing difference in the measured values for the co-/cross-polarization configurations. Importantly, the neutral excitons also efficiently scatter to the opposite valley, which, in particular, induces the initial blueshift of the resonance energy even for the cross-polarized configuration. This process is an order of magnitude faster than the free carrier scattering and occurs at the timescale of ≈240 fs consistently with other literature reports [20].

In Figure 5(b) we plot as solid lines the results of rate equations simulation. Now we also include in our model the population of neutral excitons $n_{X}^{\pm }$ and consider the following mechanisms of its relaxation: (i) intervalley scattering with $t_{nr}^{X}$ = 240 fs, (ii) trion formation process with *t*_*TF*_, (iii) radiative recombination with *t*_*X*_.

Below we present the used rate equations.

(6) $$\frac{dn_{X}^{\pm }}{dt}=\Theta_{\mathrm{Laser}}^{\pm }\left(t\right)-\frac{n_{X}^{+}-n_{X}^{-}}{t_{nr}^{X}/2}-\frac{n_{X}^{\pm }}{t_{X}}-\frac{n_{X}^{\pm }}{t_{TF}}$$

(7) $$\frac{dn_{X^{-}}^{\pm }}{dt}=\frac{n_{X}^{\pm }}{t_{TF}}-\frac{n_{X^{-}}^{\pm }}{t_{X^{-}}}$$

(8) $$\frac{dn_{e}^{\pm }}{dt}=-\frac{n_{e}^{+}-n_{e}^{-}}{t_{nr}/2}-\frac{n_{X}^{\mp }}{t_{TF}}+\frac{n_{X^{-}}^{\mp }}{t_{X^{-}}}$$

Where $n_{X}^{\pm }$ – population density of neutral excitons. Equation (6) governs the evolution of neutral excitons after their resonant creation by the pump pulse. We consider here intervalley scattering, radiative decay and charged exciton formation. In principle the formation of X^−^ is a three-particle process with its rate depending on the product of the total populations of neutral excitons and available free carriers. Here however we can simplify our model when we consider that the change of the free carrier density during the experiment is much lower than its initial value. In such a case the influence of *n*_*e*_ on this relaxation process is included through the value of the *t*_*TF*_ parameter. Equations (7) and (8), which describe temporal evolution of charged excitons and free carriers differ from the previously introduced equations (4) and (5) by the additional component of this charged exciton formation process. Measured parameters depend on the respective populations of excitons and carriers as described in the Supplementary Material (Section 4, eq. (2)–(4)), where we extend the already introduced equations (1)–(3) in order to include the effects induced by the presence of neutral excitons.

Again in the Figure 5(b) we see excellent agreement between the obtained data and simulated exciton-carrier dynamics. Interestingly, we can already note that the characteristic times of exciton decay extracted from the simulation *t*_*TF*_, *t*_*X*_ ≈ 1.2, 0.8 ps are much lower than the *t*_*X*_ = 6 ps measured in the streak camera and pump-probe experiments in the neutrality regime.

To further investigate the neutral exciton dynamics we present in the Figure 5(c) its redshift evolution for selected gate biases. As we increase the carrier density it exhibits rapid increase of its effective decay rate *t*_eff_ from which we isolate the charged exciton formation process via:

(9) $$\frac{1}{t_{\text{eff}}}=\frac{1}{t_{X}}+\frac{1}{t_{TF}}$$

In the entire range of investigated electron densities the neutral exciton decay can be estimated by fitting an exponential decay function to its redshift time evolution. These values are presented by the black curve in Figure 5(d). Moreover, in a simplified case where X^−^ decay would occur on a much longer timescale than the experimental window, one can also independently extract the ratio of *t*_*TF*_ and *t*_*X*_ times by considering the final charged exciton density (estimated e.g. from the maximum X redshift) and the initial population of photocreated neutral excitons. Here, the relaxation rates are found by simultaneous fitting of the presented model to the exciton dynamics in X/X^−^ resonant excitation (Figure 5(d)). While more general, we note that in our case this method yields values similar to the approach based on simple comparison of the estimated initial/final exciton density (see Supplementary Material). This is done for gate biases *V*_*gate*_=(0–3)V, where there is appropriately strong signal of both resonances.

For the neutrality regime at negative gate voltage we find *t*_eff_ = *t*_*X*_ = 6 ps. With the increasing density of free carriers we observe a significant shortening of the X^−^ formation time and faster X recombination which lead to the aforementioned decrease of *t*_eff_ down to ≈130 fs for carrier density of *n*_*e*_ = 2.5∗10^12^ cm^−2^.

These findings are consistent with the recent literature reports of the influence of free carriers on the neutral exciton relaxation rate [45], [46] and directly show the role of the X^−^ formation process on the overall exciton dynamics. The presence of the Fermi sea also leads to the screening of the electronic disorder, thus reducing the exciton’s inhomogeneous broadening, which is in turn related to the radiative decay rates [15], [45].

## 5 Conclusions and outlook

To conclude we presented an investigation of the ultrafast dynamics of the optical response of charged and neutral exciton complexes in monolayer MoSe_2_ and their dependence on the density of free carriers introduced by electrostatic gating. The involved temporal evolution of the optical transition lines was reliably reproduced by a simple rate equations-based model, in which we considered interactions between exciton complexes and free carriers. We showed that selective excitation of a charged exciton complex in a given valley is related to an immediate capture of a free carrier in the opposite K valley. It allows for direct optical control of the density of free carriers and provides additional path for achieving the polarization of electron gas in zero magnetic field. As such we introduce a simple and handy method for direct studies of valley-scattering mechanisms which can be easily expanded to other TMD systems. This is particularly interesting in the context of materials with different configuration of electronic bands that also exhibit a plethora of charged exciton complexes [47], [59], [63], [64], [65]. The information about their particular dynamics can be easily explored by selective excitation schemes. Additionally we directly showed how the increasing density of free carriers leads to a rapid decrease of the neutral exciton lifetime and increased rate of the trion formation channel. In the future we also aim to utilize our approach for directly probing the interaction of 2D carrier gas with an external magnetic field, where many-body interactions strongly enhance the magnetic susceptibility of this system [66].

## Supplementary Material

Supplementary Material Details

Supplementary Material Details
