# Supplementary material for: Interactions and ultrafast dynamics of exciton complexes in a monolayer semiconductor with electron gas
Source: Nanophotonics. 2024 Feb 5;13(4):487–97. doi: 10.1515/nanoph-2023-0913 (PMC11501221; doi:10.1515/nanoph-2023-0913)
Supplement: Supplementary file 1 — Supplementary Material Details [file j_nanoph-2023-0913_suppl_001.pdf]

ngerman
Research Article

Aleksander Rodek*, Kacper Oreszczuk, Tomasz Kazimierczuk, James Howarth, Takashi Taniguchi, Kenji Watanabe, Marek Potemski, and Piotr Kossacki

# Ultrafast dynamics of exciton complexes in a monolayer semiconductor interacting with the electron gas. (Supporting Information)

## 1 Exciton total absorption *versus* gate voltage

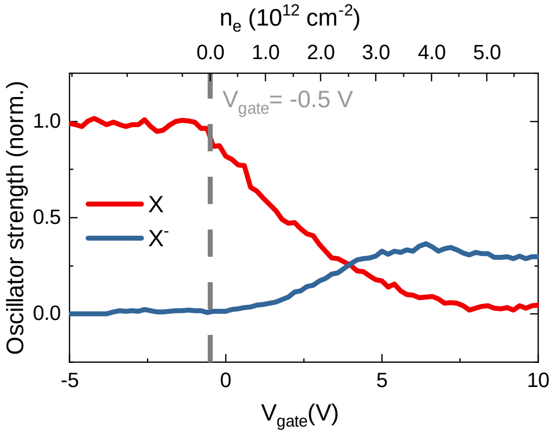


**Fig. 1: Neutral and charged exciton oscillator strengths extracted form the gate-dependence measurement.** Values were normalised by the neutral exciton intensity in the charge-neutrality regime ($V_{gate} < -0.5$ V).

***Corresponding author: Aleksander Rodek,** Faculty of Physics, University of Warsaw, ul. Pasteura 5, 02-093 Warszawa, Poland, email: aleksander.rodek@fuw.edu.pl
**Kacper Oreszczuk,** Faculty of Physics, University of Warsaw, ul. Pasteura 5, 02-093 Warszawa, Poland
**Tomasz Kazimierczuk,** Faculty of Physics, University of Warsaw, ul. Pasteura 5, 02-093 Warszawa, Poland
**James Howarth,** National Graphene Institute, University of Manchester, M13 9PL, UK
**Takashi Taniguchi,** International Center for Materials Nanoarchitectonics, National Institute for Materials Science, 1-1 Namiki, Tsukuba 305-0044, Japan
**Kenji Watanabe,** Research Center for Functional Materials, National Institute for Materials Science, 1-1 Namiki, Tsukuba 305-0044, Japan
**Marek Potemski,** Faculty of Physics, University of Warsaw, ul. Pasteura 5, 02-093 Warszawa, Poland, Laboratoire National des Champs Magnétiques Intenses, CNRS-UGA-UPS-INSA-EMFL, 25 Av. des Martyrs, 38042 Grenoble, France
**Piotr Kossacki,** Faculty of Physics, University of Warsaw, ul. Pasteura 5, 02-093 Warszawa, Poland

## 2 Streak camera measurements

In the main text we argued that for the neutrality regime of gate voltages below -0.5 V the neutral exciton energy dynamics in the pump-probe measurements exhibit a pronounced blueshift that decays on the timescale of $\tau_r \approx 6$ ps in relation to its radiative recombination. In the Fig. 2b we present the neutral exciton energy for the X-resonant pump-probe measurement in the charge neutrality regime. The data is fitted with a biexponential decay function where the shorter sub-ps relaxation is related to the intervalley scattering of neutral excitons ($\tau_{nr} \approx 400$ fs). The longer, radiative component $\tau_r$=(7.5±1) ps. This is also consistent with the streak camera measurement of the neutral X PL decay while under excitation by the Ti:Sapphire fs laser of wavelength $\lambda$=600 nm, which is presented in the Fig. 2a. Here the data is again fitted with a biexponential decay function, which is now convoluted with the gaussian profile of the temporal resolution of our setup($\sigma_t$=2 ps). The faster component is related to the exciton radiative decay and equals $\tau_r$=(6±1) ps. The "tail" of the PL signal that stretches to longer timescales comes from other relaxation processes, which may include contributions from localized or dark exciton states.

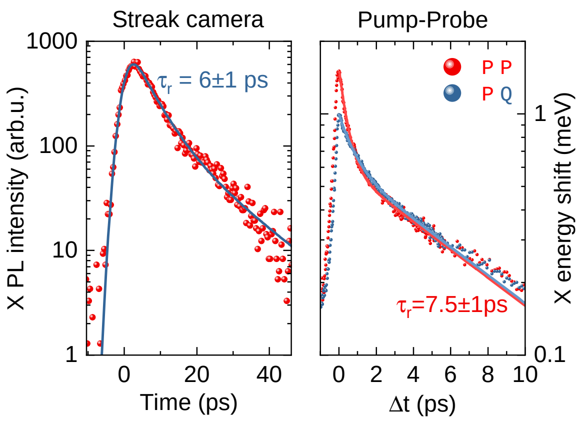


**Fig. 2:** Neutral exciton PL dynamics measured by the streak camera with the fitted decay function. Neutral exciton energy dynamics in the pump-probe measurements for a given polarization configuration with the fitted decay. $V_{gate}$=-1 V, T=5 K

In the Fig. 3 we present the results of streak camera measurements of charged exciton PL decay. In contrast to the reflection measurements from the Fig. 1 of the main text the charged exciton signal in the PL is also visible for negative gate bias, thus allowing for the extraction of its recombination time even in the neutrality regime. In the Fig. 3a we show the decay of the charged exciton PL for $V_{gate} = 1$ V with the fitted monoexponential function again convoluted with the gaussian of $\sigma_t$=2 ps, which gives the $\tau_r \approx 40$ ps recombination time used in the rate equation simulations. Additionally we are able to determine the changes of its relaxation time for higher free carrier densities (Fig. 3b).

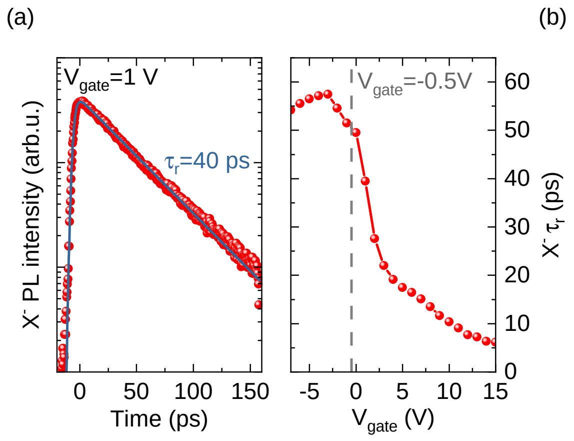


**Fig. 3:** (a) Charged exciton PL dynamics measured by the streak camera with the fitted decay function. $V_{gate}$=1 V (b) Charged exciton PL decay time *versus* gate voltage as measured by the streak camera. T=5 K

# 3 Estimation of the density of photocreated excitons.

In the pump-probe experimental setup in order to obtain the large factor of pump signal filtering its optical path is slightly misaligned with the axis of the microscopic lens. As a consequence its spot size on the sample surface is larger than the diffraction-limited area of the probe beam and, as such, the estimation of its power density may be rather challenging and requires additional geometrical parameter. In order to circumvent this problem we can calculate the density of photocreated charged excitons for a given voltage by using as a reference point the neutral exciton density, that was excited in the neutrality regime ($V_{gate}$=-1 V).

The total density of photocreated charged excitons is then given by

$$n_{X^-}(V) = \frac{A_{X^-}(V)}{A_X(-1)} * \frac{S_{X^-}(V)}{S_X(-1)} * n_X(-1) * \frac{P(V)}{P(-1)} \quad (1)$$

Where $A_{X,X^-}$—oscillator strengths of neutral and charged excitons, $S_{X,X^-}$ — spectral overlap of exciton resonances with the pump laser and $P$ — laser power, $n_X(-1)$ — density of photocreated neutral excitons in the neutrality regime. Then the neutral exciton density can by calculated from its energy shift at the coincidence, which is given by $\Delta E_X = H * n_X$. Here $H = (0.9 \pm 0.7) * 10^{-12}$(meV cm$^2$) quantifies the exciton-exciton interaction with its value taken from the literature [1]. This method only requires that the geometry of experimental setup does not change.

# 4 Rate equations calculations.

In the main text Fig. 4a we plotted as solid lines the outcome of the simulated changes in the exciton oscillator strengths and neutral exciton energy shift in the pump-probe measurement for the $X^-$ resonant excitation, where the dependence of measured parameters was related to the carrier densities through equations (1-3).

Below we also present the analogous set of equations used for describing the dynamics presented in the main text Fig. 5b. In this case the neutral exciton was resonantly excited.

$$\Delta A_X^{\pm} = -\zeta * n_X - A_X(0)[\alpha * n_e^{\mp} + \beta^X(n_{X^-}^{+} + n_{X^-}^{-})] \quad (2)$$

$$\Delta A_{X^-}^{\pm} = A_X(0)[\gamma * n_e^{\mp} - \beta^{X^-}(n_X^{+} + n_X^{-})] \quad (3)$$

$$\Delta E_X^{\pm} = H * n_X^{\pm} + \eta * n_e^{\mp} \quad (4)$$

Here $\alpha, \gamma, \eta$ are the previously introduced parameters describing the free electron influence on the exciton spectra. Similarly to previously considered case the parameters $\beta^X, \beta^{X^-}$ quantify the effects of exciton bleaching due to the population of photocreated excitons. The exciton-exciton interaction that results in the energy blueshift of neutral exciton is introduced through $H$, while $\zeta$ govern the bleaching of neutral exciton absorption by the population of photocreated neutral excitons.

In Table 1 we present the parameter values used in the simulations shown in the Fig. 4,5. Exciton redshift parameter $\eta$ can be directly compared with its value extracted in the sec. III of the main text($\eta = 0.8 \pm 0.3$). So is the case for the $\alpha$ and $\beta$, which relate to the effective change of neutral exciton osc. str. as a function of the density of photocreated charged excitons at t=0 ($\alpha, \beta = 6 \pm 2, 2 \pm 1$).

| Parameter | Pump $X^-$ | Pump X |
|---|---|---|
| Exciton density ($10^{11}$ cm$^{-2}$) | $n_{X^-} = 2$ | $n_X = 7$ |
| $\alpha$ ($10^{-13}$ cm$^{-2}$) | 7 | 7 |
| $\beta$ ($10^{-13}$ cm$^{-2}$) | 3 | 3.5 |
| $\gamma$ ($10^{-13}$ cm$^{-2}$) | 1 | 1 |
| $\eta$ ($10^{-11}$ $\frac{meV}{cm^{-2}}$) | 0.5 | 0.5 |
| $H$ ($10^{-12}$ $\frac{meV}{cm^{-2}}$) | — | 1.3 |
| $\zeta$ ($10^{-11}$ cm$^{-2}$) | — | 0.01 |
| $\beta^{X^-}$ ($10^{-13}$ cm$^{-2}$) | — | 0.1 |

**Tab. 1:** Summary of the simulation parameters for neutral and charged exciton dynamics in the pump-probe measurements

# 5 Neutral exciton resonance dependence on the charged exciton population for different electron densities

In the Fig. 4 we present the gate dependence of the parameters introduced in the main text sec.3, which describe the behavior of neutral exciton peak when we selectively excite the charged exciton. In particular, from the Fig. 4a we observe a universal redshift of the neutral exciton in the co-polarized excitation scheme (negative value of $\eta$) across the entire investigated range of gate voltages. Data shown in the Fig. 4b illustrates the effect of exciton linewidth narrowing/broadening, while in the Fig. 4c we present the parameters, which quantify the observed increase of exciton oscillator due to the decreasing population of free carriers ($\alpha$) and exciton bleaching by the created population of charged excitons ($\beta$).

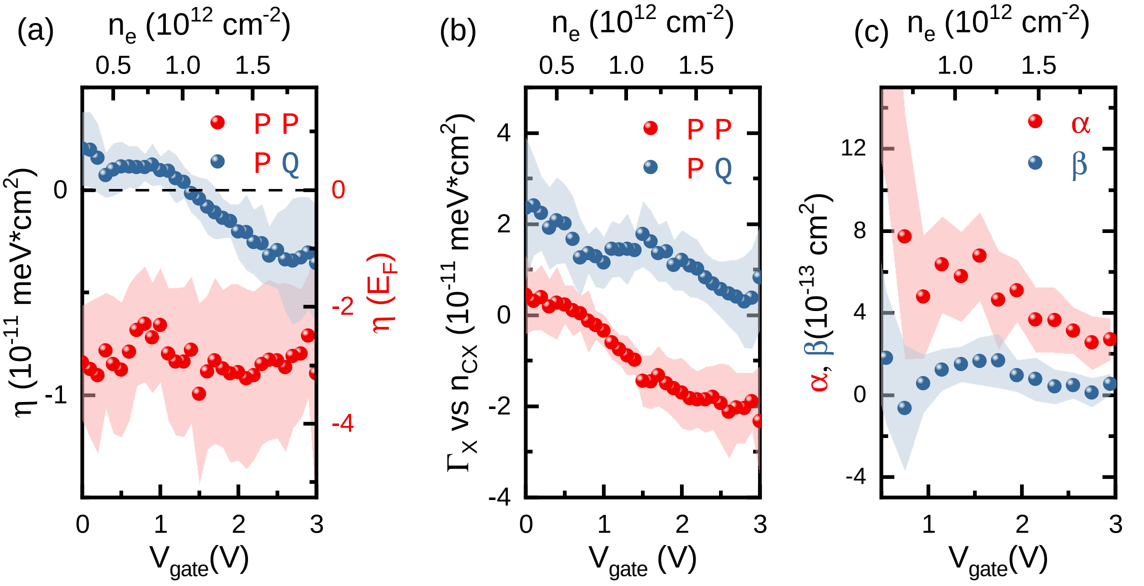


**Fig. 4:** The dependence of the effective parameter $\eta$(a), exciton linewidth(b) and $\alpha, \beta$ parameters(c) on the density of photocreated charged excitons in co-/cross-polarised valley for different gate biases.

# 6 Alternative method for the extraction of the trion formation time

In the Fig. 5d in the main text we presented the extracted values of the neutral exciton decay times related to the trion formation process and exciton recombination.

Here, in the Fig. 5 we present a comparison with an alternative approach, where one does not consider charged exciton decay. In such a case the ratio between the characteristic times of different exciton decay processes is given by:

$$\frac{t_X}{t_{TF}} = \frac{\frac{n_{X-}^{final}}{n_X(0)}}{1 - \frac{n_{X-}^{final}}{n_X(0)}} \quad (5)$$

where $t_X, t_{TF}$—exciton recombination and trion formation times, $n_X(0)$—inital density of photocreated neutral excitons, $n_{X^-}^{final}$—final density of charged excitons taken in this case at $\Delta t$=10ps. Exact values can be then calculated from the effective exciton lifetime extracted by fitting the exponential decay of neutral exciton redshift. (*eq. (9) main text*)

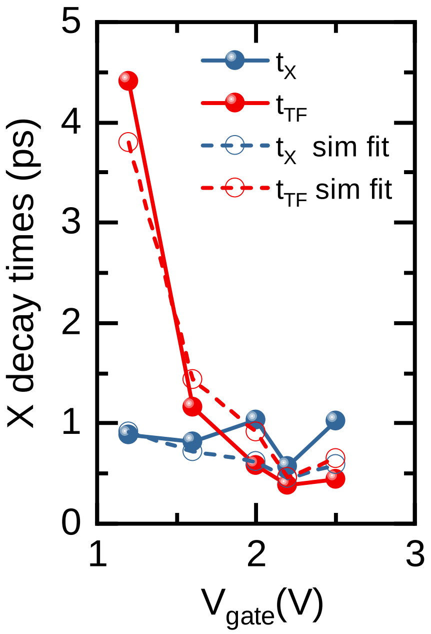


**Fig. 5:** Neutral exciton recombination and trion formation times extracted by fitting the simulated model and by estimating the initial/final densities of exciton poplations.

# References

[1] Rodek, A. *et al.* Local field effects in ultrafast light–matter interaction measured by pump-probe spectroscopy of monolayer mose2. *Nanophotonics* **10**, 2717–2728 (2021).
